# Dynamic metabolic profiles of the marine macroalga *Ulva prolifera* during fragmentation-induced proliferation

**DOI:** 10.1371/journal.pone.0214491

**Published:** 2019-05-15

**Authors:** Yanli He, Yanhui Wang, Chaoyang Hu, Xue Sun, Yahe Li, Nianjun Xu

**Affiliations:** 1 Key Laboratory of Applied Marine Biotechnology of Ministry of Education, School of Marine Science, Ningbo University, Ningbo, Zhejiang, P.R. China; 2 Zhejiang Pharmaceutical College, Ningbo, Zhejiang, P.R. China; Hainan University, CHINA

## Abstract

*Ulva prolifera*, a type of marine macroalgae, is the causative species behind green tides mainly in the Yellow Sea and adjacent regions. Nevertheless, it can be used as food or animal feed in South China. The vegetative fragments of *U*. *prolifera* are an important seed source for successive green tide blooms. Fragmentation shortens the transition time from the vegetative state to the reproductive state. However, the translation of the algal metabolites during gametogenesis is far from well understood. In this study, the dynamic metabolic profiles of *U*. *prolifera* thallus during fragmentation-induced proliferation were investigated using non-targeted metabolomics approach via a series of time course experiments in June 2017. After a 30 min low temperature shock, fragmentation induced a reproductive response of 91.57% of *U*. *prolifera* in 48 h, whereas the value was only 21.43% in the control group. A total of 156 chromatographic peaks were detected, and 63 metabolites were significantly changed in *U*. *prolifera* during reproduction. Aanlysis of the kinetic metabolic pattern showed that the fragments not only induced the formation of sporangia, but also led to complex metabolite accumulation. During fragmentation-induced proliferation, *U*. *prolifera* consumed different sugars at different time points. γ-Aminobutyric acid (GABA), glutamic acid, gallic acid, and malic acid may play important roles in germ cell formation and in the release of *U*. *prolifera*, whereas n-hexanol, 2-methyl-3-phenylindole, and 3-indoleacetonitrile may be beneficial for biotic stress resistance. Compared with the control group, in the treatment group, metabolites such as alcohols and organic acids also showed significant difference with the photoperiod at the initial stage of proliferation (before 60 h). In conclusion, changes in the levels of metabolites, including sugars, organic acids, and alcohol with photoperiod may be the strategy adopted by *U*. *prolifera* to cope with fragmentation in nature.

## Introduction

Marine macroalgae are potential sources of oil, food, and valuable compounds [1]. *Ulva* spp. are attractive models for understanding plant growth, development, and evolution, but they can cause severe oceanic green tides. *Ulva* species generally have complex life cycles, alternating between diploid and haploid generation, and exhibit parthenogenetic development of gametes. The germ cells of *Ulva* species are transformed directly from vegetative cells [2]. This transformation is caused by external factors. Tissue fragmentation is considered to be an important factor in the rapid induction of this transformation and reproduction [3]. Fragmentation significantly increases the sporulation rate of *U*. *mutabilis* from 15.8% to 80.0% [4]. Within 2–3 d after fragmentation, reproduction of *U*. *pertusa* was induced [2]. Gao et al. (2010) found that almost all *U*. *prolifera* fragments with a diameter of 0.5 mm were transformed into sporangia, whereas the sporangia in larger fragments was only formed by marginal and sub-marginal cells [5].

The mechanism of fragmentation-induction has been preliminarily considered that cutting destroyed cell walls and the additional cell-matrix structure, making it easier for algal segments to filter out inhibitors, thus removing the regulatory barriers to reproduction [2]. The gametogenesis of mature blades can be artificially induced by mincing the thallus into single monolayered fragments and by washing-out of the sporulation inhibitors [6–8]. Previous studies have showed that fragmentation-induction only improved the conversion rate of *Ulva* sporangia, and *Ulva* filaments can also be transformed into sporangia under the same culture conditions [4, 5]. Cell differentiation is a popular topic in macroalga research [9, 10]. However, the spatiotemporal changes in algal metabolites are far from well understood. Thus, we aimed to identify changes in the chemical composition of the metabolome during gametogenesis of *U prolifera*. Metabolomics is a powerful tool for the study of the metabolism and physiological functions of marine organisms including seaweed [11, 12]. Several metabolomic studies on the growth and reproduction of plants have been conducted [13]. In recent years, studies on marine macrophytes such as *Ectocarpus*, *Porphyra*, and *Zostera* species by GC-MS (gas chromatography-mass spectrometry) have provided a comprehensive understanding of the metabolic networks linked to photorespiration pathways, tricarboxylic acid cycle, glycolysis, and the pentose phosphate pathway, which are involved in the acclimatization to external stressors [13, 14]. In seaweed, aromatic amino acids (tryptophan, tyrosine and phenylalanine) and glucogenic amino acids (amino acids that can be converted into glucose via gluconeogenesis: asparagine, proline, aspartate, serine, glycine, glutamine and glutamate) have been found to be the major central organic nitrogenous compounds [15–17]. Sugars (ribulose, fructose, sucrose, trehalose) and sugar alcohols (myo-inositol, mannitol, ribitol) have been found to be other important groups of metabolites with osmoregulatory and antioxidant properties [18, 19]. In this study, the metabolites of *U*. *prolifera* in both natural reproduction and fragmentation-induced reproduction at different time points were investigated, and the results may help identify the causes of the rapid formation of marine green tides.

## Materials and methods

### Culture conditions

Vegetative *U*. *prolifera* thalli were collected from Xuwen Seaweed Co., Ltd., Xiangshan County, Zhejiang Province, China (29°05′065N, 121°56′153E), in June 2017. The thalli were gently washed three times with sterile seawater and then rinsed thoroughly. Using a magnifying glass, the attached sediments, small herbivores, and epiphytes were removed with a brush. Then, thalli were cultured in f/2 medium with the following specific parameters for acclimation for about a month before the experiments: light intensity, 70 mmol photons m^-2^ s^-1^; temperature, 25 ± 1°C; light:dark cycle, 12 h:12 h in an intelligent illumination incubator (GXZ-280 C, Ningbo Jiangnan Instruments, China); and salinity, 22 ± 1. Ampicillin (100 mg.L^-1^), gentamycin (50 mg.L^-1^) and GeO_2_ (0.3 mg L^-1^) were added to suppress the growth of bacteria and diatoms [20] The medium was aerated and refreshed every other day. Unless otherwise specified, the following experiments were performed under the same conditions. The formal experiment began after 20–25 d of domestication.

### Sample treatments

In the treatment group, *Ulva* thalli were harvested in the morning and cut into small single-layered fragments (200–500 cells per fragment) using a Zyliss Smart Clean food chopper. Two grams of fresh algal fragments was cultured in a 3000-mL flask containing 2500 mL of sterile artificial seawater. In the control group, two grams of fresh uncut vegetative thalli was cultured under the same conditions. Both groups were precultured at 4°C for 30 min in a refrigerator and performed with six duplicates. Samples (0.2 g) were collected at culture times of 12, 24, 36, 48, 60, 72, 96, and 120 h, and the media volume was decreased about 250 mL to maintain the same culture density.

### Microscopic observation and counting methods

The formation and release of *U*. *prolifera* germ cells were observed under an AOTE BK5000 microscope equipped with a Cool SNAP digital camera system. Seven microscopic fields of view were randomly observed in each selected fragments or uncut thalli. During the maturation process, the germ cell sporangia and released germ cell sporangia were imaged for counting. The percentage of mature germ cell sporangia and the formation and release rates were calculated using the following equations: Sp = (Sm /At)×100% and Rp = (Ss/At)×100%, where S_p_ represents the percentage of mature sporangia, S_m_ represents the mature sporangia, R_p_ represents the percentage of released germ cell cysts per thalli, S_s_ represents the germ cell cysts that were released (in μm^2^), and A_t_ represents the number of germ cell cysts in the whole field under the microscope.

### Metabolite extraction and GC-MS analysis

#### Extraction

Sample materials (20±2 mg) were put in 2-mL Eppendorf tubes. One metal or zirconia ball was added in every tube. Seven hundred microliters of 100% methanol and 5.75 μL of stock ribitol (Sigma-Aldrich, München, Germany, 0.2 mg/mL stock in water) were added, and the tubes were then vortexed for 15 seconds. Followed by homogenization with a Retschmühle instrument for 3 min at 25 Hz. The tubes were then centrifuged for 10 min at 14000 rpm and 600 μL of the upper phase was transferred into 2-mL fresh Eppendorf tubes, then 300μL CHCl_3_ was added and mixed slowly. Then, 750 μL H_2_O was added, and the tubes were vortexed for 15 seconds. Subsequently, the tubes were centrifuged for 10 min at 14000 rpm, and 150 μL of the upper phase (polar phase) was taken into 1.5-mL Eppendorf tubes. Finally, all the extracts were dried in a speed vac for at least 3 h under ambient temperature conditions.

#### Derivatization

After evaporation of the solvent, 40 μL of methoxyamine hydrochloride (Sigma-Aldrich) solution (20 mg/mL) in pyridine was added for derivatization, and the mixture was incubated at 60°C for 1 h. Subsequently, 50 μL of N-methyl-N-(trimethylsilyl) trifluoroacetamide (Macherey-Nagel, Düren, Germany) was added to each sample and the samples were incubated at 40°C for 1 h. Then, transferred 100 μL was transferred into sample vials and analyzed directly by GC-MS.

#### GC-MS analysis

The metabolites were detected using a Trace 1300/ITQ 900 GC-MS system (Thermo Fisher Scientific, USA, Serial No.13005). GC-MS analysis was conducted as previously reported [21] with slight modifications. The samples were injected with a 1300 autosampler equipped with a 10-μL tapered, fixed-needle, polytetrafluorethylene (PTFE)-tipped plunger syringe. The column and all spare parts were purchased from Thermo Fisher Scientific (Thermo Fisher Scientific, USA). The GC parameters for the analysis were as follows: The inlet temperature was 250°C; the The EI ion source temperature was 230°C, high-purity helium (99.99%) as the carrier gas, and 1 μL of sample was injected with the splitless mode. The temperature was initially set at 60°C for 2 min, then increased by 10°C min^−1^ up to 150°C and held for10 min. Then, the temperature was increased to 250°C at 15°C min^−1^ and held for 20 min. MS was performed with a full-scan mode with a mass range of 30 to 550 (m/z). Peak area extraction was performed with the Thermo Scientific software Xcalibur (Thermo Fisher Scientific Inc., Waltham, MA, USA). The relative metabolite content was calculated by an internal-standard-based normalization method.

#### Data analysis

Compounds were identified based on the ratio and relative abundance of the National Institute of Standards and Technology database (NIST) and MassBank website (http://www.massbank.jp/) according to mass/charge. Statistical analyses were performed in MetaboAnalyst 3.0 (http://www.metaboanalyst.ca/) according to its protocol (Linting and Meulman 2011). “Normalization by internal standard ribitol” was chosen for sample normalization, “None” for data transformation, and “Auto scaling” for data scaling. Principal component analysis (PCA) and orthogonal partial least squares discriminant analysis (OPLS-DA) were performed in the SIMCA-P 13.0 software package using auto scaling. MATLAB 7.5 was selected to build a heat map diagram with Pearson parameters via the hierarchical clustering algorithm, the other parameters took the default values. Two-sided t-test, one-way ANOVA, two-way ANOVA, post-hoc analysis, and ANOVA-simultaneous component analysis (ASCA) were performed in MetaboAnalyst 3.0 (http://www.metaboanalyst.ca/) according to its protocol[22]. “Auto scaling” was selected as the data scaling method for all analyses. The two-way ANOVA type used was “within subject ANOVA.” Statistical significance was considered at *P* < 0.05, and the false discovery rate was used for multiple testing correction. For ASCA, the alpha and leverage thresholds were set as 0.05 and 0.9, respectively. Figures were edited with Adobe Illustrator CS5 for improved resolution.

## Results

### Microscopic observation of the germ cell sac formation

**Fig 1:**
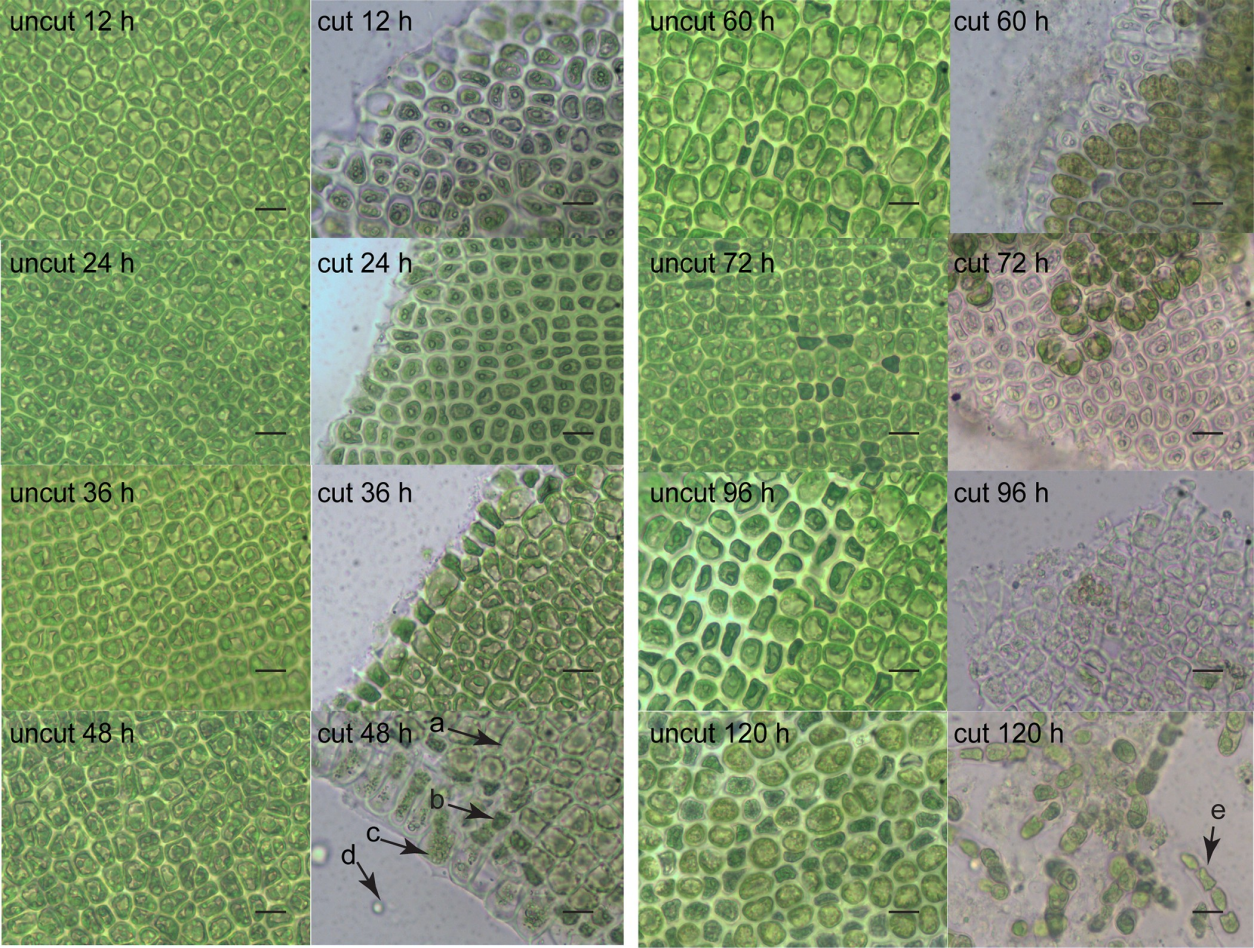
Microscopic observation of the transformation of *U*. *prolifera* from the vegetative state to the reproductive state after cutting. Left: uncut(control); right: cut (treatment) 12, 24, 36, 48, 60, 72, 96, and 120 h. For 48 h of treatment: a, b, c, d, represent vegetative cell, mature germ cell sac, released germ cell sac, and released gametophytes/sporophytes respectively, for 120h of treatment: e represents germinated seedling, the scale bars represent 20 μm.

As shown in Fig 1, the mature germ cell sac first appeared on the edge of the cut algae at 24 h, but most of the cells remained in a vegetative state at 60 h in the control group. At 48 h, nearly all of the vegetative cells (91.57%) (Table 1) in the treatment group were transformed into germ cell sacs, and the germ cell sacs that matured early at the edge began to be release. However, only 9.57% of the vegetative cells were transformed into germ cell sacs at 60 h in the control group (Table 1). At 72 h, nearly all of the vegetative cells (98.29%) were transformed into mature germ cell sacs in the treatment group, while 20.43% of the algae were transformed into germ cells (Table 1) in the control group. At 96 h, almost all of the cells were transformed into mature germ cell sacs. Moreover, germinated seedlings appeared in the treatment group. However, only 4.28% of the germ cell sacs were released in the control group although most of the vegetative cells were transformed into germ cell sacs (120 h, Fig 1).

**Table 1:** Transformation of *U*. *prolifera* from vegetative cells into germ cell sac and their release rate.

	Time (h)	12	24	36	48	60	72	96	120
*S*p (%)^a^	Treated groups	0	15.71^c^	21.43	91.57	97	98.29	100	100
	Control groups	0	0	12.86	21.43	32.86	95.29	98.57	98.43
*R*p (%)^b^	Treated groups	0	0	0	4.14	9.57	20.43	41.29	96.29
	Control groups	0	0	0	0	0	0	0	4.28

^a^percentage of mature germ cell sporangia

^b^release rates of germ cell sporangia

^c^average of six tests

### Analysis of metabolite profiles

**Fig 2:**
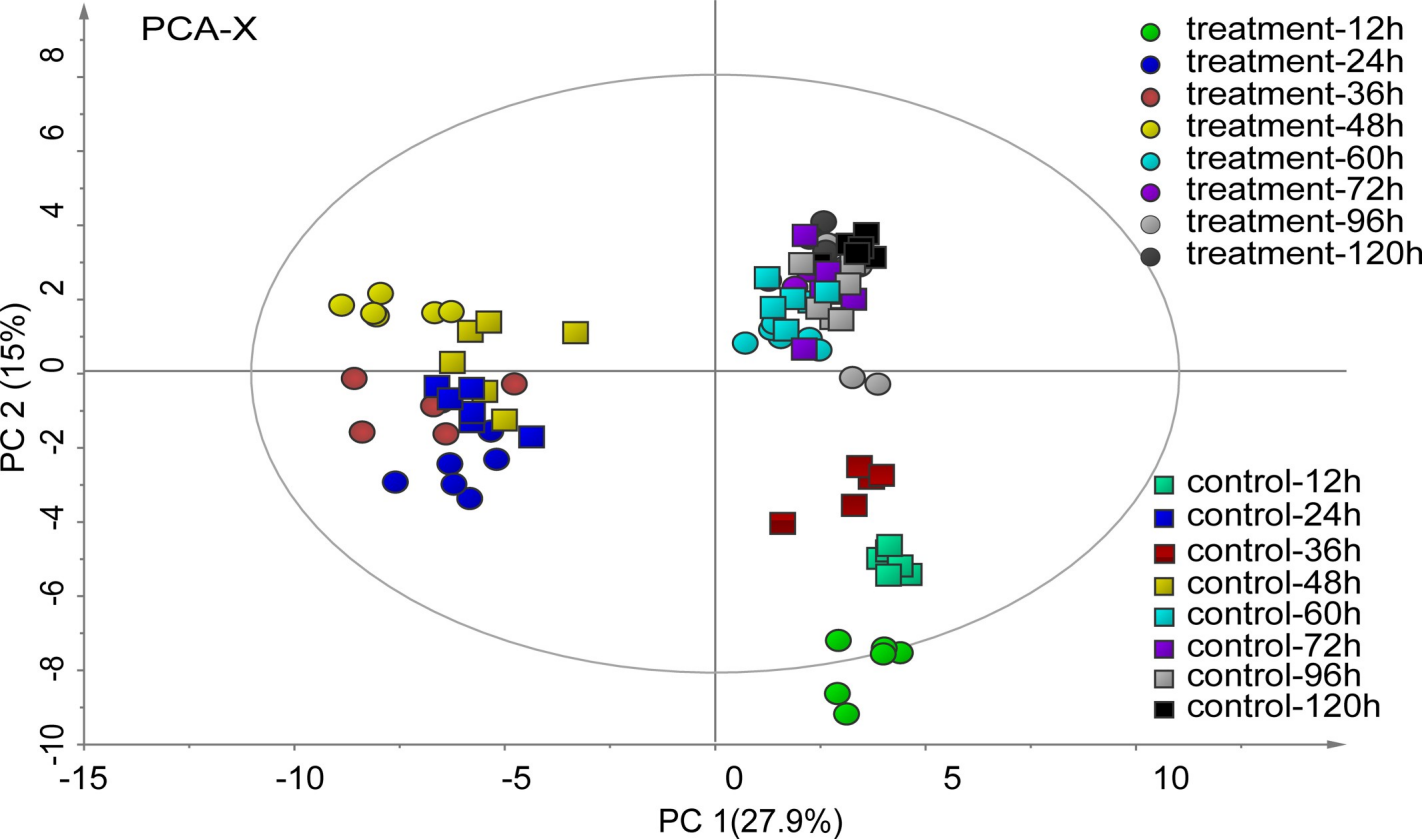
PCA of *U*. *prolifera* during its fragmentation-induced proliferation. The green, dark-blue, brown, pale-yellow, pale-blue, purple, gray and black colors represent treatment the 12, 24, 36, 48, 60, 72, 96, and 120 h treatments respectively, and boxes (□) and circles (○) represents the control and treatment groups, respectively; n = 6.

To investigate the metabolic profiles of *U*. *prolifera* during fragmentation-induced proliferation, both the filament and fragments at 12, 24, 36, 48, 60, 72, 96, and 120 h were collected and subjected to metabolic profiling analysis based on GC-MS. Smoothing and baseline correction were performed prior to statistical analysis. The baseline, area noise and peak noise were set at 400, 20 and 10, respectively. After background subtraction, the extraneous peaks due to solvent, column material loss or impurities from the sample preparation process were removed, and only peaks with m/z values with signal-to-noise ratio more than 10 were selected. By this process, 156 chromatographic peaks were detected. The GC-MS data were eventually organized into two-dimensional matrices, including observed values (samples) and variables (peak intensities). PCA was subsequently performed on the 156 metabolites to obtain overall patterns within the kinetic metabolic patterns and induction methods for *U*. *prolifera* reproduction. The first principal component (PC1), accounting for 27.9% of the total variance, reflected time-dependent *U*. *prolifera* reproduction, and samples from the treatment and control groups were clustered at the same time point (Fig 2). The results indicated that the developmental time affected *U*. *prolifera* metabolites more than the induction method did. The second principal component (PC2) accounted for 15% of the total variance. The samples of the treatment and control groups at the same time points were all distinguishable in the PCA plots, indicating that the metabolites with different treatments of *U*. *prolifera* were different at the same developmental times.

**Fig 3:**
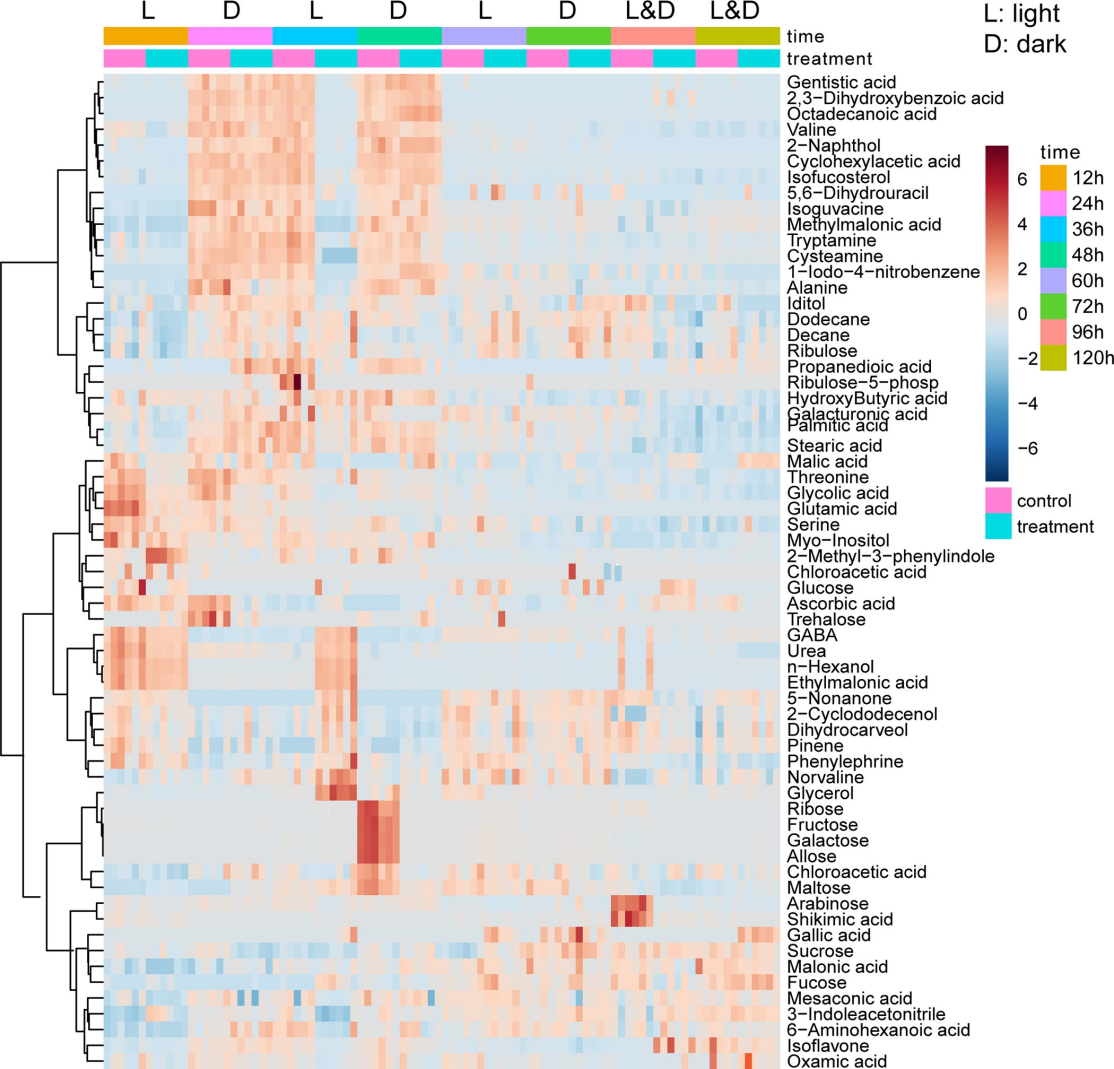
Heatmap analysis, combined with a hierarchical cluster analysis (HCA), of the metabolites in *U*. *prolifera* fragmentation-induced proliferation (treatment and control groups) during the 12–120 h period (n = 6). In the first line “L” represents light and “D” represents dark; In the second/time line colors represent different time; in the third/treatment line, the color pink represents the “control group” and green represents the “treatment group”.

PCA provides a high-level summary of the main patterns of data variance. Detailed metabolite abundance profiles can be obtained by visualization of metabolomic data based on heat map. The changes of metabolites in the cut and uncut thalli over time were almost the same. Notably, these metabolites exhibited opposite trends in the treatment and control groups at the 36 and 48 h time points (Fig 3).

**Fig 4:**
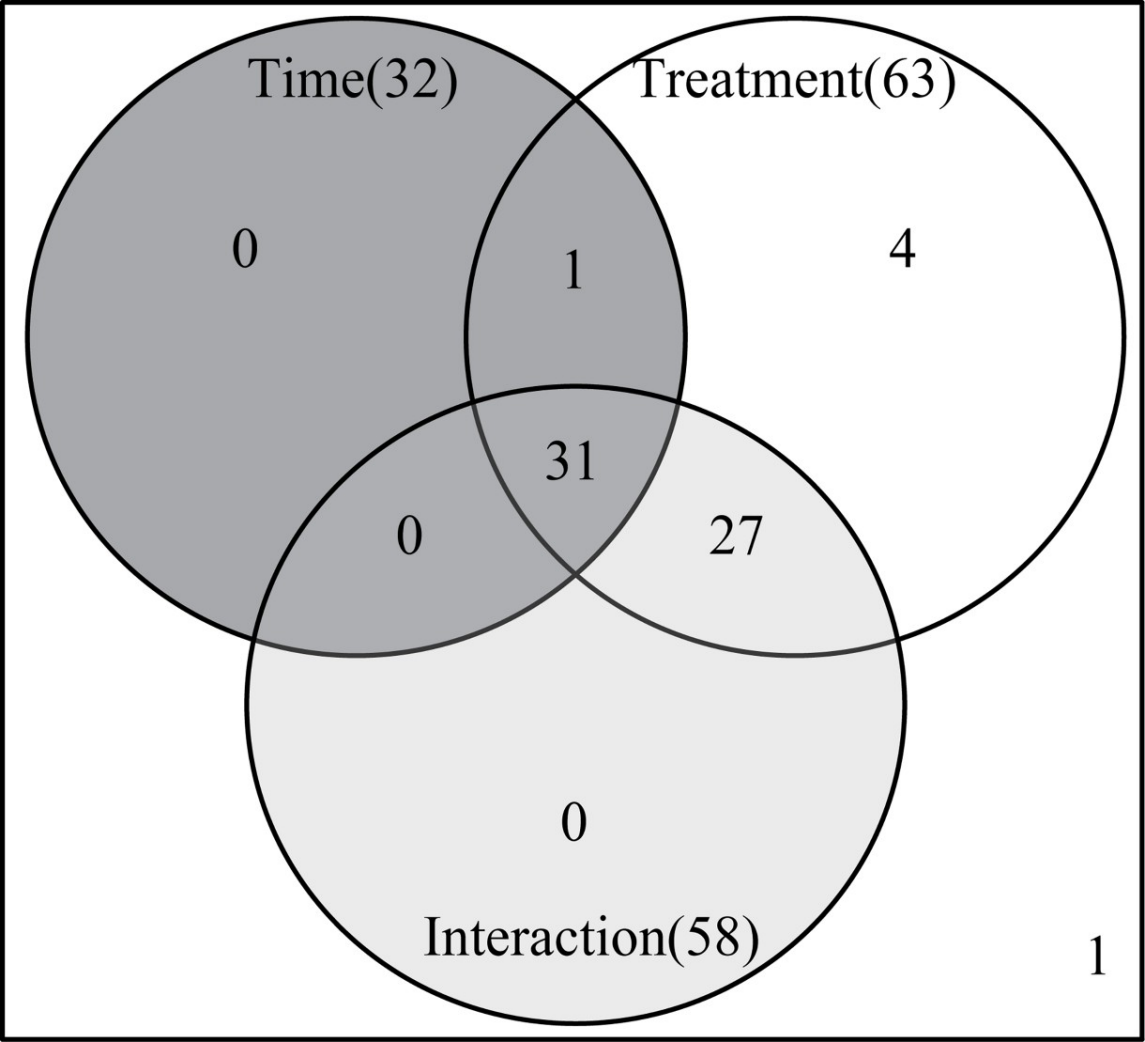
Two-way repeated measures (within subjects) ANOVA of metabolites in *U*. *prolifera* fragmentation-induced proliferation during 12–120 h. Pink, purple, and green circles respect time, treatment, and their interaction, respectively; the figures in the circles represent the kinds of metabolites that responded to the element. The following parameters were chosen: ANOVA type, “Type ǀ”; Consider interactions, “Yes”; Adjusted p-value cutoff, 0.05; Multiple testing correction, false discovery rate.

Two-way repeated measures (within subjects) ANOVA was then utilized to analyze which the factors (treatment, developmental time, and their interaction) that caused the variation of each metabolite. The abundances of 63, 32, and 58 metabolites were affected by treatment, developmental time, and their interaction, respectively (Fig 4).

**Fig 5:**
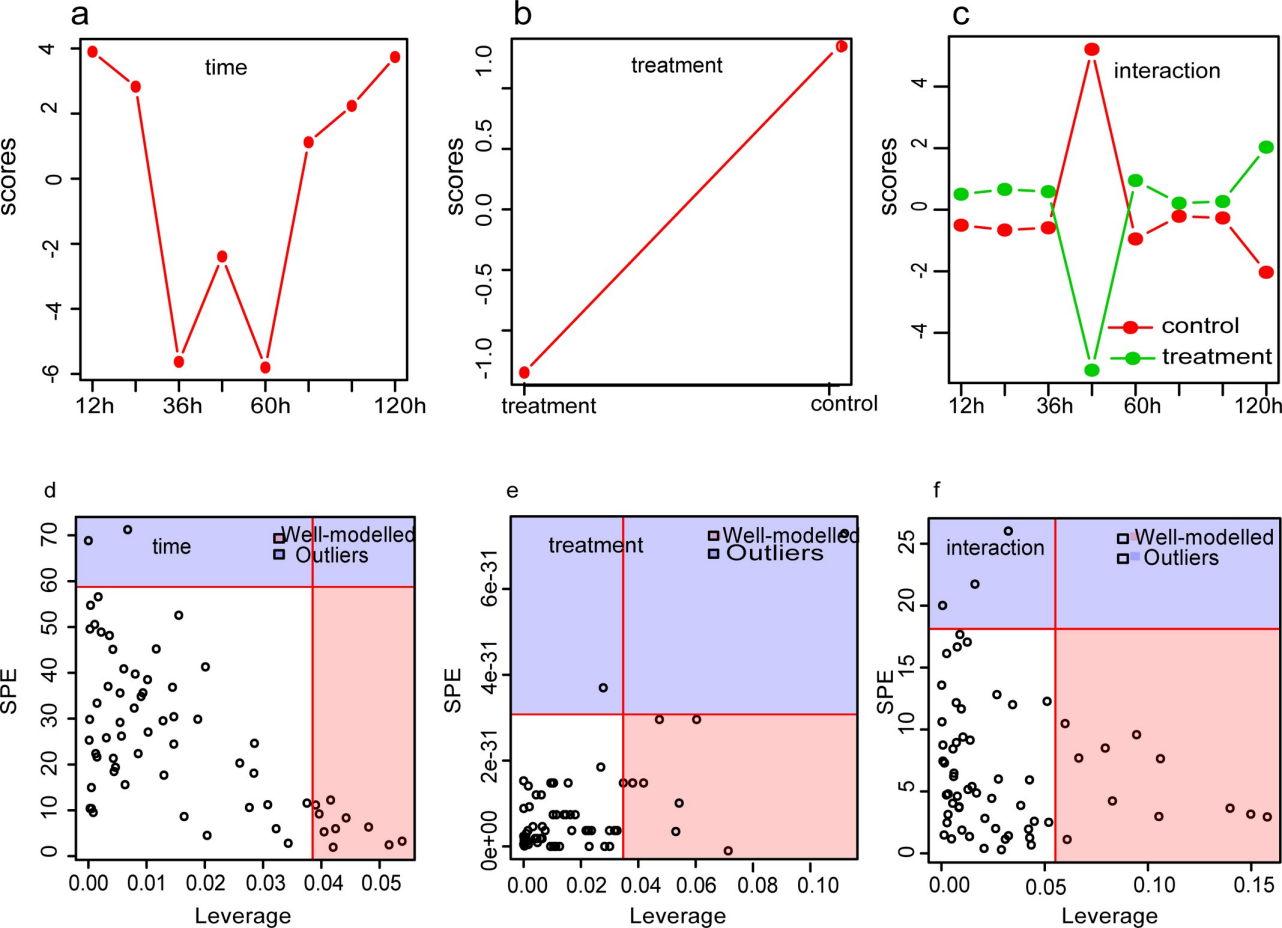
ANOVA-simultaneous component analysis (ASCA) of *U*. *prolifera* during fragmentation-induced proliferation. (a-c) Major pattern associated with time, treatment and their interaction; (d-f) ASCA selection of important variables (metabolites) associated with time, treatment and their interaction by leverage/SPE analysis. The list of well-modeled metabolites refer to Table 2.

ASCA was performed to determine the trends associated with treatment method, developmental time, and their interaction patterns [22]. The time score plots based on component 1 (45.21% of variation explained) of the corresponding model demonstrated a decrease in scores from 12 to 36 h, an increase at 48 h, a decrease from 48 to 60 h, and a final increase until 120 h (Fig 5A). The treatment score plot showed that different treatment types differed in their PC1 scores, with the treatment and control groups exhibiting the lowest and highest scores, respectively (Fig 5B). Component 1 of the interaction effect exhibited clear opposite trends at 48 h between the treatment and control groups, while the same trend was observed at other times (Fig 5C).

Leverage/squared prediction error (SPE) plots were made to correlate metabolic features with experimental factors [22]. The leverage evaluates the importance of the metabolite to the model, and SPE tests the fitness of the model for particular metabolites. Metabolites with high-leverage SPEs, which contribute significantly to the model, were chosen as well-modeled metabolites. By this method, 63 metabolites that were affected by treatment were analyze by SPE, a total of 31 metabolites were well modeled, including 3 alcohols, 5 amino acids, 11 organic acids 6 sugars and 6 other kinds of metabolites (Table 2). Twelve well-modeled metabolites, including six organic acids (cyclohexylacetic acid, gentisic acid, octadecanoic acid, 2,3-dihydroxybenzoic acid, stearic acid, palmitic acid), two alcohols (isofucosterol and 2-naphthol), one amino acid (valine) and two other kinds of metabolites (tryptamine and 5-nonanone), were determined based on the major time pattern (Fig 5D and Table 2). The levels of these metabolites decreased sharply from 0 h to 24 h in the filament and its fragments. Then, the lecels increased at 36 h, decreased at 48 h, and peaked at 96 h. Eight metabolites, namely glycolic acid, glycerol, alanine, valine, arabinose, norvaline, chloroacetic acid allyl ester, and ribose, were well modeled by treatment method (Fig 5E and Table 2). Eleven metabolites, including fructose, allose, ribose, glycerol, cysteamine, galactose, ribulose-5-phosphate, ascorbic acid, 3-indoleacetonitrile, methylmalonic acid, and malic acid, were well modeled (Fig 5F and Table 2) based on the interaction, and their levels varied between the filament and its fragments.

**Table 2:** Metabolites of *U*. *prolifera* which met the following criteria: potential biomarker according OPLS-DA, well-modeled by SPE and with Hotelling’s T2 value > 100.

Name	Hotelling-T2	time(F.val)	time(raw.p)	time(adj.p)	phynotype(F.val)	phynotype(raw.p)	phynotype(adj.p)	Interaction(F.val)	Interaction(raw.p)	Leverage	SPE
Malic acid^3^^,^^4^	8.65E+02	3.43E+00	9.39E-02	1.43E-01	4.67E+00	2.44E-04	2.70E-04	9.33E+00	8.29E-08	5.35E-02	1.29E+01
Glycerol^3^^,^^4^^,^	7.83E+02	1.14E+02	8.64E-07	6.27E-06	9.52E+01	3.28E-33	1.75E-32	8.21E+01	3.58E-30	1.20E-01	2.26E+00
Octadecanoic acid^1^	7.65E+02	9.89E+00	1.04E-02	2.47E-02	3.71E+02	1.05E-52	3.37E-51	8.15E+01	3.78E-30	4.43E-02	8.35E+00
GABA^3^^,^^4^	5.06E+02	6.36E+00	3.03E-02	5.70E-02	5.23E+01	2.57E-25	7.83E-25	2.26E+01	4.42E-15	5.25E-02	1.96E+00
2,3-Dihydroxybenzoic acid^1^	4.97E+02	6.67E+00	2.73E-02	5.29E-02	1.63E+02	9.48E-41	2.02E-39	3.70E+01	2.72E-20	4.16E-02	1.23E+01
Maltose^3^^,^^4^	4.53E+02	1.64E+01	2.32E-03	8.25E-03	8.53E+01	1.01E-31	4.62E-31	3.57E+01	5.82E-20	5.43E-02	7.56E+00
Palmitic acid^1^	4.42E+02	5.64E+00	3.89E-02	6.73E-02	3.41E+01	3.74E-20	8.26E-20	4.47E+00	5.41E-04	3.90E-02	1.12E+01
Urea^4^	4.30E+02	1.02E-02	9.21E-01	9.21E-01	5.87E+01	8.73E-27	2.94E-26	1.63E+01	4.59E-12		
1-Iodo-4-nitrobenzene^1^^,^^4^	3.97E+02	1.21E+01	5.98E-03	1.82E-02	3.54E+01	1.32E-20	3.25E-20	9.54E+00	6.15E-08	3.61E-02	7.51E+00
Glycolic acid^2^^,^^4^	3.53E+02	1.86E+02	8.69E-08	4.46E-06	7.26E+01	1.42E-29	5.70E-29	1.22E+01	1.00E-09	1.02E-01	1.47E+00
Glucose^4^	3.18E+02	9.35E-01	3.56E-01	4.30E-01	3.73E+00	1.75E-03	1.86E-03	2.61E+00	2.35E-02		
n-Hexanol^4^	3.13E+02	2.17E+00	1.71E-01	2.24E-01	5.96E+01	5.47E-27	1.95E-26	1.92E+01	1.57E-13		
Cyclohexylacetic acid^1^	3.07E+02	1.68E+02	1.41E-07	4.46E-06	5.40E+02	2.83E-58	1.81E-56	9.67E+01	2.53E-32	5.39E-02	3.27E+00
Methylmalonic acid^4^	3.06E+02	9.24E+00	1.25E-02	2.85E-02	5.53E+01	4.99E-26	1.60E-25	2.34E+01	2.07E-15		
Ascorbic acid^3^^,^^4^	2.85E+02	2.69E+00	1.32E-01	1.88E-01	2.70E+01	1.45E-17	2.72E-17	1.69E+01	2.23E-12	5.98E-02	3.98E+00
Valine^1^^,^^2^^,^^4^	2.82E+02	1.14E+02	8.82E-07	6.27E-06	1.04E+02	2.06E-34	1.32E-33	1.35E+01	1.76E-10	4.24E-02	5.99E+00
Cysteamine^3^^,^^4^	2.75E+02	4.52E+01	5.23E-05	2.79E-04	8.17E+01	3.93E-31	1.68E-30	5.46E+01	5.75E-25	6.82E-02	2.83E+00
Ethylmalonic acid	2.58E+02	2.39E+00	1.53E-01	2.13E-01	6.22E+01	1.51E-27	5.69E-27	1.82E+01	4.70E-13		
Isofucosterol^1^^,^^4^	2.57E+02	7.92E+00	1.83E-02	3.79E-02	1.51E+02	1.30E-39	1.67E-38	2.07E+01	3.19E-14	4.20E-02	1.95E+00
Alanine^2^	2.53E+02	4.52E+01	5.23E-05	2.79E-04	3.48E+01	2.22E-20	5.09E-20	7.93E+00	8.70E-07	4.17E-02	2.95E+00
Arabinose^2^^,^^4^	2.45E+02	1.44E+02	2.90E-07	4.46E-06	4.74E+01	4.25E-24	1.24E-23	5.29E+01	1.30E-24	4.94E-02	0.00E+00
Propanedioic acid ester^4^	2.44E+02	2.62E-01	6.20E-01	6.50E-01	3.26E+01	1.21E-19	2.50E-19	2.63E+01	1.34E-16		
Glutamic acid^4^	2.05E+02	1.14E+02	8.62E-07	6.27E-06	1.40E+02	1.35E-38	1.11E-37	3.51E+01	8.10E-20		
Allose^3^^,^^4^	1.92E+02	1.08E+02	1.13E-06	7.26E-06	1.44E+02	6.11E-39	6.52E-38	1.45E+02	1.41E-37	1.37E-01	2.64E+00
Gentistic acid^1^^,^^4^	1.88E+02	1.72E+01	1.99E-03	7.51E-03	9.06E+01	1.55E-32	7.62E-32	1.35E+01	1.69E-10	4.81E-02	6.36E+00
Ribose^2^^,^^3^^,^^4^	1.79E+02	1.45E+02	2.85E-07	4.46E-06	1.40E+02	1.38E-38	1.11E-37	1.41E+02	2.66E-37	3.46E-02	2.95E+00
Galactose^3^^,^^4^	1.77E+02	1.39E+02	3.49E-07	4.46E-06	1.57E+02	3.54E-40	5.67E-39	1.55E+02	3.13E-38	1.37E-01	2.74E+00
Fructose^3^^,^^4^	1.56E+02	1.30E+02	4.73E-07	5.05E-06	1.36E+02	4.01E-38	2.85E-37	1.36E+02	5.24E-37	1.36E-01	2.63E+00
5-Nonanone^1^	1.48E+02	5.84E+00	3.63E-02	6.45E-02	3.14E+01	3.24E-19	6.49E-19	2.04E+01	4.32E-14	3.75E-02	1.16E+01
Chloroacetic acid allyl ester^2^	1.44E+02	1.17E+01	6.50E-03	1.89E-02	7.82E+00	5.72E-07	7.18E-07	3.88E+00	1.75E-03	3.52E-02	2.95E+00
2-Naphthol^1^^,^^4^	1.39E+02	3.01E+01	2.67E-04	1.22E-03	9.80E+01	1.33E-33	7.72E-33	1.91E+01	1.57E-13	5.16E-02	2.48E+00
Gallic acid^2^^,^^4^	1.13E+02	2.16E+01	9.17E-04	3.91E-03	8.68E+00	1.25E-07	1.67E-07	4.24E+00	8.44E-04	5.88E-02	1.47E+00
3-Indoleacetonitrile^3^^,^^4^	7.33E+01	4.43E+00	6.17E-02	1.01E-01	1.39E+01	3.63E-11	5.53E-11	1.06E+01	1.06E-08	5.64E-02	6.13E+00
Norvaline^2^^,^^4^	5.36E+01	7.33E+00	2.20E-02	4.41E-02	6.24E+00	1.06E-05	1.22E-05	7.35E-01	6.53E-01	3.55E-02	2.96E+00

^1^metabolites well-modeled with time.

^2^metabolites well-modeled with treatment.

^3^metabolites well-modeled with interaction.

^4^potential biomarkers according to OPLS-DA.

### Metabolic difference analysis

**Fig 6:**
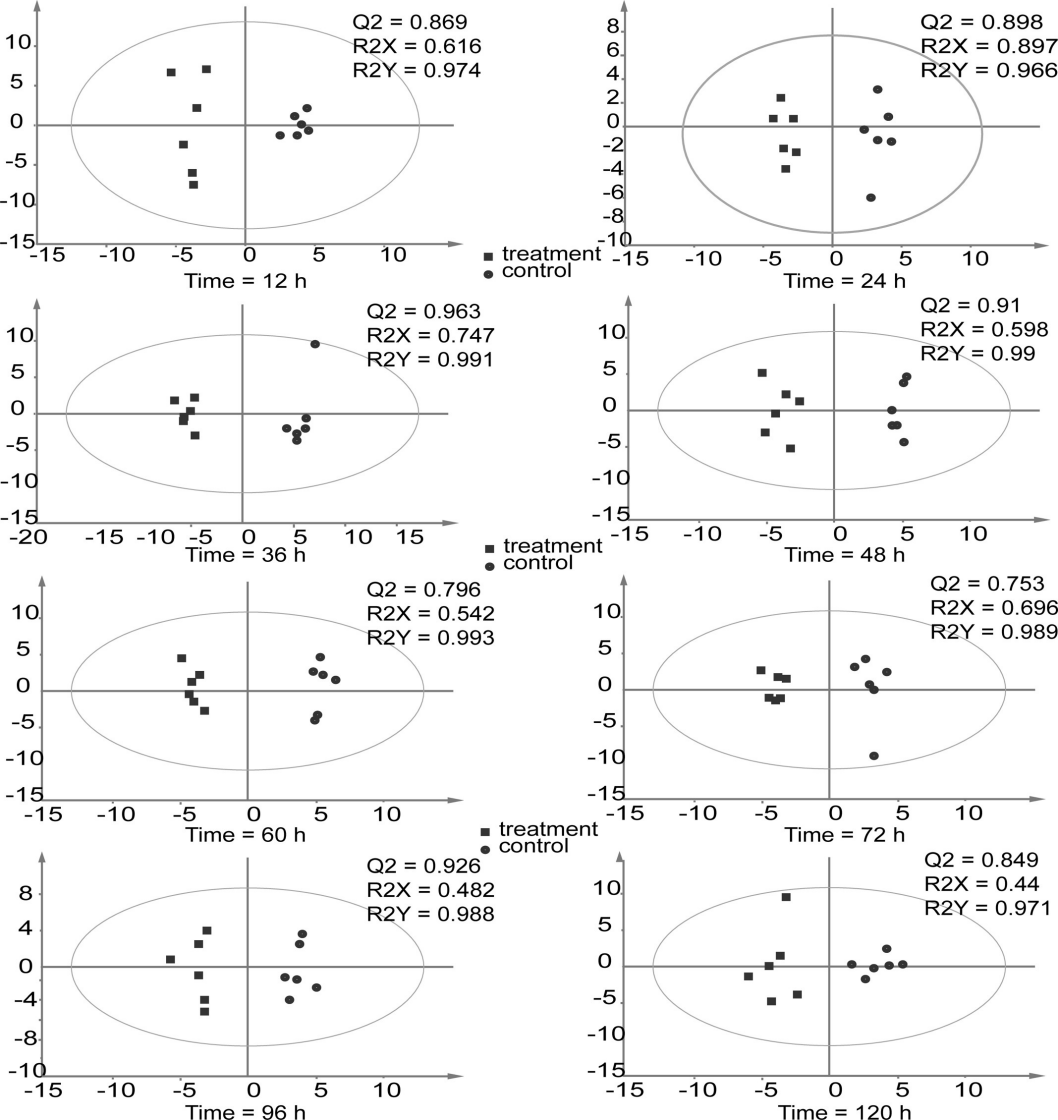
OPLS-DA of the treatment (cut) and control (uncut) groups of *U*. *prolifera* at different time points. UV scaling was selected; ■ and● represent the treatment(cut) and control (uncut) groups, respectively.

To simplify the information and obtain maximal covariance among metabolite levels during proliferation at different time points, OPLS-DA was applied. The OPLS-DA model, which was composed of one orthogonal component and one prediction component, generated the explained variation values R2X (cum) > 0.82 (except at 96 and 120 h where the R2X values were 0.482 and 0.44) and R2Y (cum) > 0.966 and the predictive capability Q2 (cum) > 0.753 at all six time points (Fig 6). These high-value parameters indicated the excellence in modeling and prediction with good discrimination between the fragmentation-induced proliferation and natural proliferation of *U*. *prolifera* at each time point before 96 h because the model parameters (>0.5) are considered to be satisfactory in explanatory and predictive capabilities [23].

Based on a variable importance in projection (VIP) threshold (VIP > 1) from the 200-fold cross-validated OPLS-DA models, a number of metabolites associated with the distinction of metabolic profiles of fragmentation-induced proliferation and natural proliferation at each time point were procured. The candidate metabolites from the OPLS-DA model, with *P*-values are less than 0.05 between fragmentation-induced proliferation and natural proliferation at each time point tested using the T-test with a fold threshold of > 2 or < 0.5, were further selected as biomarkers (Table 3).

**Table 3:** Metabolic difference between cut and uncut groups of *U*. *prolifera* at each time point.

*RT* (min)	Name	*P*-value^a^	-log10(p)	FDR	FC^b^	VIP^c^
**12 h**^**d**^						
28.55	Maltose	8.16E-06	5.09	1.20E-04	12.23	1.75
6.58	2-Methyl-3-phenylindole	3.32E-04	3.48	3.92E-03	2.78	1.61
9.13	3-Indoleacetonitrile	4.71E-03	2.33	3.03E-02	2.33	1.41
18.3	Glutamic acid	7.86E-06	5.1	1.20E-04	0.35	1.75
**24 h**						
28.55	Maltose	2.64E-07	6.58	1.61E-05	12.44	2.02
13.41	Propanedioic acid ester	9.23E-06	5.03	1.88E-04	6.13	1.95
32.3	Trehalose	9.48E-04	3.02	9.64E-03	0.27	1.72
**36 h**						
5.87	n-Hexanol	3.39E-08	7.47	5.26E-07	15.09	1.35
10.19	5-Nonanone	8.30E-05	4.08	3.03E-04	11.26	1.24
12.14	GABA	5.09E-07	6.29	3.95E-06	6.34	1.33
12.55	Ethylmalonic acid	1.23E-06	5.91	7.66E-06	4.35	1.32
29.65	Pinene	4.98E-04	3.3	1.40E-03	4.13	1.17
5.39	Urea	5.59E-06	5.25	2.67E-05	2.67	1.3
10.01	Glycolic acid	1.23E-04	3.91	4.03E-04	0.5	1.23
28.25	Isofucosterol	1.12E-05	4.95	4.96E-05	0.44	1.29
13.41	Propanedioic acid ester	1.24E-04	3.91	4.03E-04	0.32	1.23
16.07	Methylmalonic acid	4.07E-07	6.39	3.95E-06	0.29	1.33
9.71	Isoguvacine	1.90E-05	4.72	7.85E-05	0.28	1.28
9.4	Valine	4.96E-07	6.3	3.95E-06	0.28	1.33
13.62	Ascorbic acid	5.35E-06	5.27	1.63E-04	0.24	1.96
8.72	1-Iodo-4-nitrobenzene	7.20E-08	7.14	8.93E-07	0.21	1.35
17.22	Gentisic acid	3.34E-06	5.48	1.73E-05	0.19	1.31
10.94	Succinic acid	6.67E-09	8.18	1.38E-07	0.15	1.36
30	2-Naphthol	1.49E-06	5.83	8.42E-06	0.13	1.32
13.35	Cysteamine	6.37E-07	6.2	4.39E-06	0.09	1.33
48 h						
13.91	Malic acid	2.02E-03	2.7	1.10E-02	9.61	1.43
13.62	Ascorbic acid	5.80E-03	2.24	2.32E-02	6.05	1.33
28.55	Maltose	4.20E-05	4.38	3.60E-04	0.39	1.63
10.18	Glycerol	8.77E-07	6.06	1.05E-05	0.18	1.72
25.44	Allose	2.52E-07	6.6	5.05E-06	0.05	1.74
25.51	Galactose	1.70E-07	6.77	5.05E-06	0.05	1.75
25.41	Ribose	1.80E-07	6.74	5.05E-06	0.04	1.74
20.54	Fructose	3.43E-07	6.46	5.14E-06	0.03	1.74
60 h						
10.18	Glycerol	1.62E-05	4.79	1.02E-03	2.13	2.11
18.3	Glutamic acid	4.32E-03	2.36	3.89E-02	0.45	1.72
23.61	Arabinose	5.53E-04	3.26	9.52E-03	0.38	1.92
72 h						
28.55	Maltose	8.45E-04	3.07	4.51E-02	0.49	1.99
5.78	n-Hexanol	1.48E-03	2.83	4.51E-02	0.26	1.94
90 h						
13.91	Malic acid	2.32E-03	2.64	1.49E-02	5.22	1.39
30.37	Norvaline	3.99E-03	2.4	2.10E-02	4.77	1.34
5.48	Isoflavone	2.68E-03	2.57	1.55E-02	3.37	1.38
25.4	Glucose	1.13E-03	2.95	1.09E-02	2.82	1.44
21.71	Fucose	9.68E-03	2.01	3.62E-02	0.41	1.25
10.19	5-Nonanone	6.89E-05	4.16	2.00E-03	0.29	1.58
22.77	Iditol	2.31E-03	2.64	1.49E-02	0.27	1.39
25.41	Ribose	9.68E-03	2.01	3.62E-02	0.26	1.25
28.55	Maltose	1.04E-03	2.98	1.09E-02	0.14	1.45
23.61	Arabinose	1.23E-02	1.91	3.96E-02	0.11	1.22
28.94	Shikimic acid	1.55E-02	1.81	4.49E-02	0.09	1.19
**120h**						
12.71	Gallic acid	2.57E-06	5.5905	4.96E-05	7.07	2.17
13.91	Malic acid	2.25E-08	7.6474	1.31E-06	7.02	2.25
5.39	Urea	6.53E-08	7.185	1.89E-06	0.35	2.23

^a^ *P*-value was calculated by T-test (two-tails) with a cutoff of 0.05.

^b^ Ratio of relative metabolites at 24 and 12 h after cutting.

^c^ VIP was obtained by OPLS-DA with a threshold of 1.0.

^d^ Comparison of relative content of metabolites in *U*. *prolifera* at each time point.

At 12 h, four metabolites (maltose, 2-methyl-3-phenylindole, 3-indoleacetonitrile, and glutamic acid) were selected as biomarkers (Table 3). The levels of maltose (12.23-fold), 2-methyl-3-phenylindole (2.78-fold), and 3-indoleacetonitrile (2.33-fold) were significantly higher, and the relative abundance of glutamic acid was significantly lower, than that in the control group.

At 24 h, three metabolites, maltose, propanedioic acid ester, and trehalose, were important for distinguishing between the treatment and control groups (Table 3). In the treatment group, the relative contents of trehalose decreased significantly, however, the relative content of maltose increased by 12.44-fold, and those of propanedioic acid ester also increased significantly.

At 36 h, a total of 18 metabolites, including six kinds of acids, two kinds of alcohol, one benzene derivative, one kind of lipid, six flavonoids, three lipids, two carbohydrates, and six other kinds of metabolites, played important roles in separating the treated and control groups. In the treatment group, the top five metabolites with the highest fold values were n-hexanol, 5-nonanone, GABA, ethylmalonic acid, and pinene. In particular, the relative contents of n-hexanol was 15.09-fold higher than that of the control group. The levels of five metabolites, namely, 1-iodo-4-nitrobenzene, gentisic acid, succinic acid, 2-naphthol, and cysteamine, were significantly decreased in the control groups, with cysteamine exhibiting the lowest level (0.09-fold).

At 48 h, eight metabolites, including six carbohydrates and two acids, were selected as biomarkers (Table 3). Among these metabolites, the relative levels of four kinds of monosaccharides (galactose, ribose, allose, and fructose) and glycerol was 0.39–0.03-fold lower in the treatment group than in the control group. The levels of malic acid and ascorbic acid increased significantly by 9.61and 6.05-fold, respectively. However, the levels of maltose, glycerol, allose, galactose, ribose, and fructose decreased.

At 60 h, the levels of three metabolites, namely, glycerol, arabinose, and glutamic acid, changed significantly between the treated and control group. Their relative levels of these metabolites all decreased in the fragmentation-induced proliferation groups. At 72 h, only two metabolites (maltose and n-hexanol) were selected as biomarkers.

However, at 90 h, four metabolites, namely, malic acid, norvaline, isoflavone, and glucose, accumulated significantly in the treatment group. Moreover, the relative levels of seven kinds of metabolites, namely, arabinose (0.11-fold), maltose (0.14-fold), ribose (0.26-fold), fucose (0.42-fold), shikimic acid (0.09-fold), iditol (0.27-fold), and 5-nonanone (0.29-fold), sharply decreased.

At 120 h, in the treatment group, the levels of malic acid and gallic acid increased, and that of urea decreased, compared with those in the control group.

### Kinetic metabolic pattern

**Fig 7:**
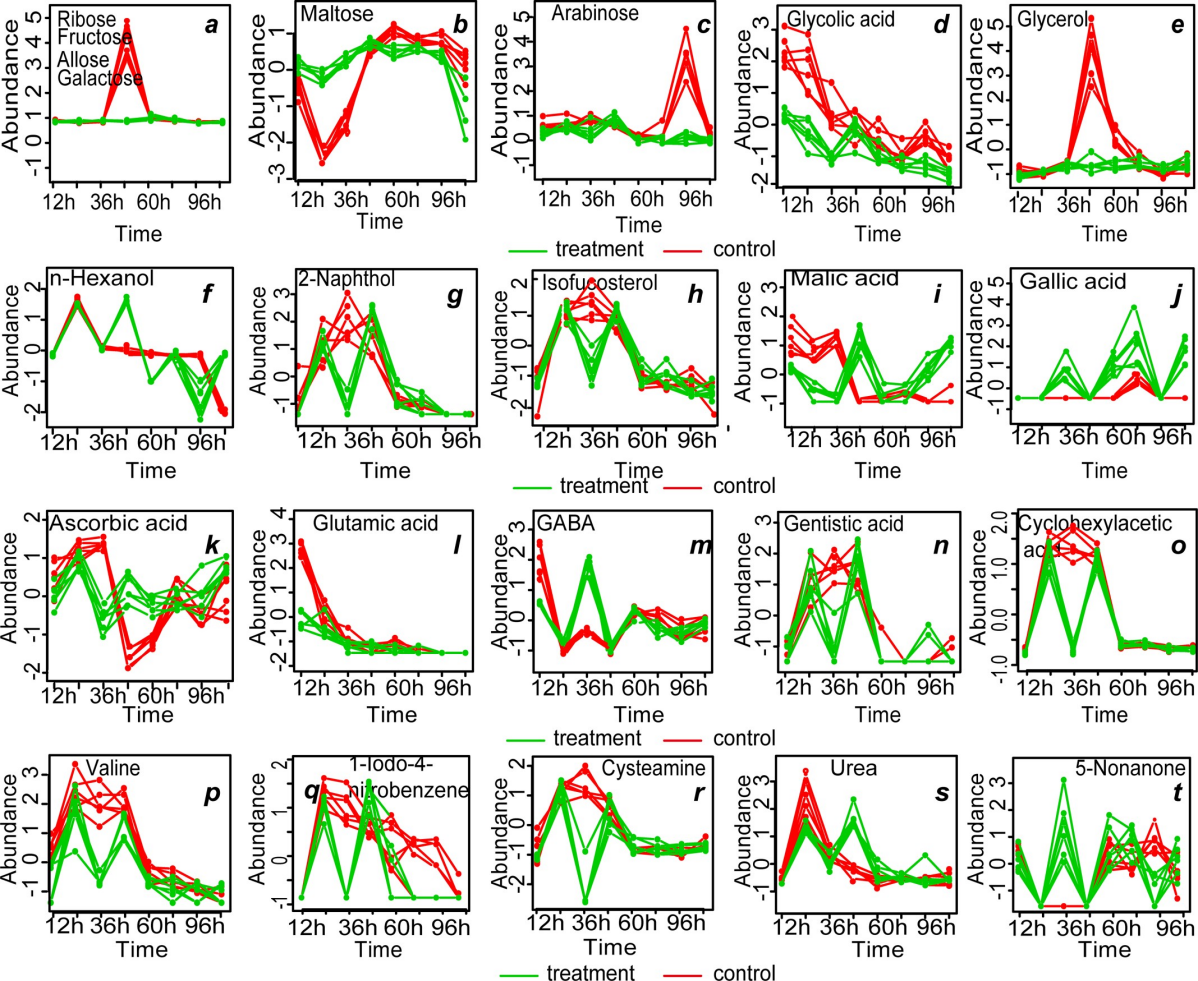
MEBA of *U*. *prolifera* metabolites that met the following criteria: potential biomarkers according OPLS-DA, well-modeled by SPE and with Hotelling’s T2 value > 100.

In order to investigate the variations of the 31 metabolites that were well modeled by SPE, multivariate Empirical Bayes Analysis (MEBA), a time-course analytical method based on the MEBA statistic, which can evaluate the importance of features by Hotelling’s T, was employed to identify the metabolites, with differential temporal profiles [24]. Twenty-three metabolites, which meet the following criteria are shown in Fig 7: potential biomarkers (Table 3), Hotelling’s T2 value > 100 and well-modelled according the analysis by ASCA are shown in Fig 7 (n-hexanol, cyclohexylacetic acid, alanine and urea varied obviously with time but did not meet the above three criteria) (Table 2). The changes in four carbohydrate metabolites (ribose, fructose, allose, and galactose) with time were consistent: from 0 to 36 h, the abundances of these metabolites varied between -1 and 0 and almost reached 4 at 48 h in the control group. Then, the abundance sharply decreased to nearly 0 at 72 h, while that in the treatment group was relatively stable (Fig 7A). This result was consistent with the heat map. For example, the abundance of maltose in the control group decreased from 0 to -2.5 and then gradually increased, peaking at 60 h, then fluctuation slightly with the photoperiod and maintaining a value between 0 and 1 at 0–48 h. The change trend was the same as that in the treatment group. However, the abundance of maltose in the treatment group fluctuated slightly between 0 and 1 with the photoperiod at 48–96 h and then decreased to less then 0 at 96–120 h (Fig 7B and Table 2). In the treatment group, the relative levels of the four alcohols were associated with the photoperiod before 60 h, and the relative content increased at night (without light) and decreased during the day (with light), after 60 h, the relative content gradually leveled off. In the control group, however, 36 h was the cutoff point, where the relative content increased before 36 h and then decreased gradually (except for that of glycolic acid, the relative level of which decreased gradually from 12 h). Interestingly, the relative levels of six other metabolites, including three organic acids (gentistic acid, ascorbic acid and cyclohexyacetic acid), valine, 1-iodo-4 nitrobenzen and cysteamine also showed this trend.

## Discussion

Fragmentation-induced spores play a vital role in the rapid accumulation of large amounts of biomass and may be one of the most important factors in the acceleration of the occurrence of green tides on the coast of Qingdao, China [5]. In this study, reproductive cells (nearly 90%) (Table 1) appeared within 48 h after *U*. *prolifera* thalli were cut into approximately 0.5-mm segments. This observation was consistent with the results of the study on *U*. *mutabilis* [4]. Interestingly, almost all the cells (96.29%) in the physiological state transformed into the reproductive state at 120 h in the control group. This finding indicated that fragmentation-induction was not a determinant of *U*. *prolifera* sporangium formation but only a promoter. The metabolic profiles of the treatment group differed significantly from that of the control group according to PCA and OPLS-DA. Time-course analysis showed that the trends for the relative levels trend of carbohydrates, alcohols and organic acids were different between the treatment and control groups (Fig 7).

### Response of different sugars during germ cell formation

Sugar is mainly involved in the biosynthesis of sugar polymers, including starch and cellulose, generation of energy (ATP), cross-talk in invertase-mediated sugar signaling, and phytohormone control [25]. The monosaccharides content in the treatment group was significantly lower than that in the control group at two time points 48 h (ribose, fructose, allose, and galactose) and 96 h (arabinose) (Figs 3 and 6), and the results were consistent with the kinetic metabolic pattern analysis (Fig 7A). Meanwhile, the maltose content was higher in the treatment group within 12–48 h after cutting and then became similar to that in the control group (Fig 7B). The result indicated that in the processes of fragmentation-induction and natural proliferation, *U*. *prolifera* consumed different sugars. Kaplan and Guy (2004) indicated that the resultant maltose accumulation may function as a compatible-solute stabilizing factor in the chloroplast stroma in response to acute temperature stress [26]. In this study, the accumulation of maltose suggests that it was not only related to energy metabolism during the spore formation of *U*. *prolifera*, but also related to the response to mechanical injury.

### Organic acids may be involved in the redox function and osmotic regulation of *U*. *prolifera*

Compared with the control group, accumulation of several organic acids was observed, including ethylmalonic acid (4.35-fold) at 24 h, malic acid (9.61-fold) and ascorbic acid (6.06-fold) at 48 h, and gallic acid (7.07-fold) and malic acid (7.02-fold) at 120 h (Table 3). During the process of transformation from vegetative to reproductive state, the pigmentation deepened, the vacuole becomes smaller and then disappeared at 36 h in the treatment and 48–60 h in the control (Fig 1). Accumulation of organic acids may contribute to the regulation of the cell osmotic pressure to facilitate the change from the vegetative to reproductive state. Likewise, Alsufyani et al. (2017) investigated the composition of the metabolites released during Ulva growth under normal conditions with an exometabolomic approach and found that higher concentrations of divalent low-molecular-weight metabolites such as maleic and succinic acids were detected in the late gametogenesis phase. Elevated levels of organic acid are important for energy production, cellular membrane stabilization, maintenance of turgor, vitrification of the cytoplasm and signaling in cells under stress conditions [13, 27]. Moreover, ascorbic acid is one of the major antioxidant compounds synthesized in plants and is used for regulation of cell division [28, 29]. In orthodox seeds, there is no ascorbic acid and ascorbic acid peroxidase activity in the stationary phase, but the activity restarts after the onset of imbibition [30]. Many studies have shown that ascorbic acid biosynthesis and rapid restart of ascorbic acid peroxidase activity are key to seed germination [31, 32]. However, other studies have shown that high doses of ascorbic acid can inhibit the germination of wheat seeds [33]. In this study, 21.43% germ cells were formed at 36 h, and 91.57% germ cells were formed at 48 h, and the germ cells started to be released in the treatment group. Furthermore, in the treatment group, the relative content of ascorbic acid decreased (0.24-fold, at 36 h point) and then increased (6.06-fold, at 48 h point) compared with those of the control group. In contrast to higher plants, reduced ascorbic acid content in *U*. *prolifera* may contribute to the formation of germ cells, and high concentrations of ascorbic acid may contribute to the release of germ cells.

Accumulation of GABA is believed to be a redox regulatory mechanism in a variety of terrestrial plants, such as *Arabidopsis* [34], *Lotus japonicus* [35], *Pisum sativum* [34, 36] and *Glycine max* [37]. Gallic acid and its related compounds showed significant chromosomal influence characteristics [38, 39]. In this study, the relative levels of GABA and gallic acid increased at 36 h in the treatment group, which was consistent with the results of Singh et al.(2016), indicating that these two organic acids play a conserved role in *U*. *prolifera* and higher plants [28, 38–41]

### Other metabolites involved in the formation of germ cells in *U*. *prolifera*

Metabolites constitute phytochemical defense weapons produced by a variety of metabolic pathways and can be broadly divided into three categories, namely, isoprenoids (e.g., diterpenes), alkaloids (e.g., indole alkaloid camalexin), and shikimates (e.g., flavonoids) [42]. In this study, at 12 h, 2-methyl-3-phenylindole and 3-indoleacetonitrile accumulated in the treatment group (Table 3), suggesting that these metabolites were used for biological defense by *U*. *prolifera*.

Two o alcohol metabolites: glycerol and hexanol exhibited distinct changes (Table 3). The content of glycerol in the treatment group was significantly higher than that in the control group at 60 h after cutting (Fig 7E). In this study, the Sp of *U*. *prolifera* was 97% and the Rp was 9.57% at 60 h after cutting, but the values were only 32.86% and zero, respectively, at 60h in the control group. Alsufyani et al. (2017) found that the maximum content of glycerol on the surface of *Ulva* was observed at 21 h after artificial induction, and the glycerol secreted by *U*. *prolifera* might be the carbon source for its epiphytes. In this experiment, the relative content of glycerol increased significantly at 60 h (Fig 7E), which might be the result of the interaction between *U*. *prolifera* and the epiphytes which was beneficial for the formation of the germ cells [43]. Hexanol, a green leaf volatile that can be used as a synergist for insect pheromones, is used for controlling insect populations. In this study, the content of hexanol was affected by not only cutting, but also photoperiod. The hexanol content at night (24, 48, and 72 h) was significantly higher than that in the daytime (12, 36, and 60 h), indicating that hexanol might protect *U*. *prolifera* from harm caused by nocturnal insect (Fig 7F). Certainly, further targeted screening for organic acids is necessary to understand the functions of fragmentation-induced proliferation.

Compared with previous studies that focused on metabolites at a specific time point [44, 45] or target metabolites [46–48], this study examined the changes in metabolites at different time points during sporangium formation of *U*. *prolifera*, and the dynamic metabolomic changes that occur during cell differentiation were discussed. After cutting, the reproductive sporangia were formed earlier than those in the control group, and various metabolites underwent drastic changes. The kinds of consumed sugars differed significantly at different time points during the formation of sporangia. At the initial stage of proliferation (the first 60 h), the metabolites such as alcohol and organic acid showed significant changes with the photoperiod, which may be the main strategy by which *U*. *prolifera* copes with fragmentation in nature. However, the GC-MS approach provided a very preliminary view of the metabolic changes that were the consequences or the causes of the differentiation of reproductive cells. Identification of effective biomarkers to monitor spatiotemporal changes in complex cellular processes was only the first step, and the challenging isolation and structural elucidation of the compounds have to be performed in further studies. In addition, bacterial morphogenetic compounds might not be detected among these metabolites, as the biologically active concentrations of these compounds [49–51] seem to be far below the detection limit of the metabolomic approach applied [43]. As (epiphytic) bacteria are essential for *Ulva* morphogenesis, bacterial compounds are certainly part of the surface-associated metabolome. Future studies will explore these compounds by comparison, e.g., axenic *Ulva* cultures with normally developed adult axenic cultures grown in the presence of purified morphogenetic bacterial compounds [43].
